# The impact of diabetes mellitus and other chronic medical conditions on health-related Quality of Life: Is the whole greater than the sum of its parts?

**DOI:** 10.1186/1477-7525-3-2

**Published:** 2005-01-12

**Authors:** Hwee-Lin Wee, Yin-Bun Cheung, Shu-Chuen Li, Kok-Yong Fong, Julian Thumboo

**Affiliations:** 1 Department of Pharmacy, National University of Singapore, 18 Science Drive 4, Singapore 117543, Republic of Singapore; 2 Department of Rheumatology and Immunology, Singapore General Hospital, Outram Road, Singapore 169608, Republic of Singapore; 3 National Cancer Centre Singapore, 11 Hospital Drive, Singapore 169610, Republic of Singapore; 4 Department of Medicine, National University of Singapore, 10 Medical Drive, Singapore 117597, Republic of Singapore

## Abstract

**Background:**

Diabetes mellitus (DM) is an important public health concern, the impact of which is increased by the high prevalence of co-existing chronic medical conditions among subjects with DM. The aims of this study were therefore to (1) evaluate the impact of DM and co-existing chronic medical conditions on health-related quality of life (HRQoL) (which could be additive, synergistic or subtractive); (2) to determine the extent to which the SF-6D (a single-index preference measure) captures the multidimensional information provided by the SF-36 (a profile measure).

**Methods:**

Using data from a cross-sectional, population-based survey of Chinese, Malay and Indians in Singapore, we developed 9 separate multiple linear regression models, with each SF-36 scale or SF-6D index score being the dependent variable for one model. The influence of DM and a second chronic medical condition (hypertension (HTN), heart disease (HD), musculoskeletal illnesses (MS)) and their interactions were studied after adjusting for the influence of potential confounding variables.

**Results:**

Among 5,224 subjects, the prevalence of DM, HTN, HD and MS were 5.9%, 10.7%, 2.4% and 26.6% respectively. DM lowered SF-36 scores by more than 2 points on 3 SF-36 scales and lowered SF-6D scores by 0.03 points. Subjects with DM and HTN, DM and HD or DM and MS experienced further lowering of SF-36 scores exceeding 2 points on at least 6 scales and further lowering of SF-6D scores by 0.05, 0.08 and 0.10 points respectively. Generally, DM and co-existing medical conditions exerted additive effects on HRQoL, with the exception of DM and heart disease, where a subtractive effect was noted. SF-6D index scores generally reflected the patterns of influence of DM and chronic medical conditions on SF-36 scores.

**Conclusion:**

DM and chronic medical conditions generally reduced HRQoL in this multiethnic general population in an additive, rather than synergistic or subtractive fashion. In this study, the SF-6D was a reasonably good summary measure for the SF-36.

## Background

Diabetes mellitus, the prevalence of which is reaching epidemic proportions in many parts of the world, is an increasingly important public health concern. In the United States, diabetes is present in 8% of the adult population, and is associated with a two-fold increase in age-adjusted mortality [1,2]. In addition, there is a high prevalence of chronic medical conditions among subjects with diabetes [3-5]. For example, in the United States, the prevalence of cardiovascular diseases, stroke and depression in subjects with diabetes was at least twice as high as in subjects without diabetes [6-9].

Diabetes has detrimental effects on a range of health outcomes including health-related quality of life (HRQoL) [10-12]. For example, in the Medical Outcomes Study, diabetes was found to impair all dimensions of health except mental health and pain [13]. In a more recent multinational study, diabetes was found to have a notable impact on general health, measured using the Medical Outcomes Short-Form 36 (SF-36) [14]. The magnitude of impact of diabetes on HRQoL was reported to be equivalent to that of having cardiovascular conditions, cancer and chronic respiratory disease [15].

Subjects with diabetes and multiple co-existing chronic medical conditions have poorer HRQoL than those without these conditions [16,17]. For example, subjects with diabetes and co-existing cardiovascular diseases reported significantly lower scores on RAND-36 social functioning, vitality and health-change scales [16]. In another study, subjects with diabetes and co-existing coronary artery disease, peripheral sensory neuropathy and peripheral vascular diseases reported significantly lower scores on several SF-36 scales [17].

**Figure 1:**
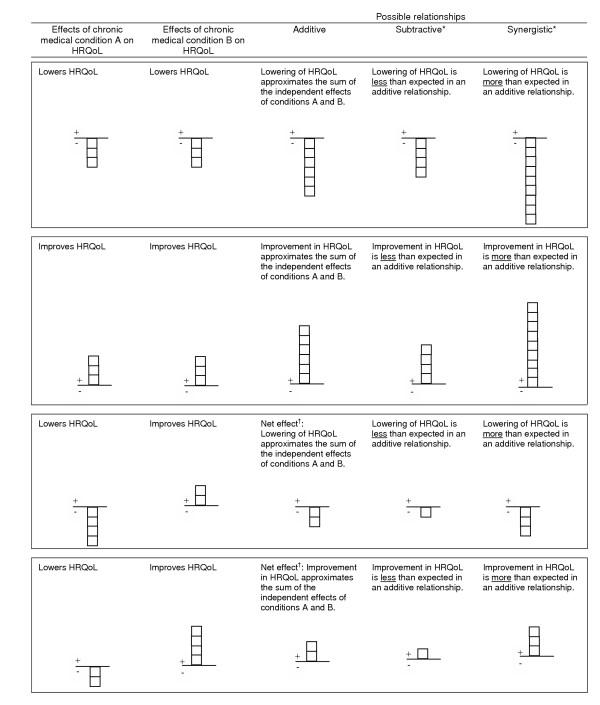
An overview of the possible relationships among multiple chronic medical conditions on HRQoL. *These relationships (subtractive or synergistic) are discussed in relation to additive relationships. ^†^If net effect is zero, then discussion of subtractive and synergistic relationships do not apply.

In combination, the influence of multiple chronic medical conditions on HRQoL may exhibit an additive, synergistic or possibly subtractive relationship. Assuming that each chronic medical condition results in the lowering of HRQoL, in an additive relationship, the combined effect of two or more chronic medical conditions on HRQoL approximates the sum of the independent effect of each of these conditions, while in a synergistic relationship, the combined effect is greater than the sum of the independent effect of each of these conditions and in a subtractive relationship the combined effect is smaller than the sum of the independent effect of each of these conditions (Figure 1). For example, the SF-36 developers [18] reported an additive relationship between hypertension and other co-existing chronic medical conditions on HRQoL, while Gaynes et al. [19] reported synergistic relationships between depression and co-existing arthritis on physical functioning and between depression and co-existing diabetes on role functioning. Several mechanisms could account for these observed synergistic effects. For example, treatment for one medical condition might also adversely affect another pre-existing medical condition, leading to a greater lowering of HRQoL than would occur due to the pre-existing condition alone [20]. Additionally, a medical condition itself (e.g. depression) might adversely affect patient behavior and thus negatively affect treatment outcomes [21]. Although there have not been any reports in the literature, it is theoretically possible that subtractive relationships exist. For example, it was reported that patients undergo changes in their conception of poor levels of functioning, personal values (e.g. changes in life priority) and/or meaning of life in response to their chronic medical conditions, a concept known as response shift [22]. These changes in self-assessment and values may help to cushion the impact of the second medical condition, resulting in a smaller decrement in HRQoL than might otherwise be expected.

Two published studies have evaluated the relationship between diabetes and other chronic medical conditions on HRQoL in the general population [23,24]. In the first study [23], diabetes and stroke were found to increase the risks of disability (measured with the Activity of Daily Living and Instrumental Activity of Daily Living scales) among older Mexican Americans in an additive fashion. In the second study, the impact of co-existing obesity, hypertension or diabetes and heart disease on HRQoL among 17,195 U.S. middle- and older-age adults was evaluated [24]. A strong synergistic relationship between heart disease and diabetes on the odds of mobility difficulty (1.8 to 4.0 times), activity of daily living limitations (2.2 to 4.0 times) and poor perceived health (2.1 to 6.8 times) was found, after adjusting for gender, ethnicity, insurance status, history of cancer, and lung diseases [24]. However, there were two important limitations in both studies. First, these studies focused on the physical domains of HRQoL, without assessing the mental and social domains of HRQoL. Second, both studies were conducted among middle-aged and/or elderly adults. Thus, the burden of diabetes and other chronic medical conditions on HRQoL in the rest of the general population with diabetes are not known. Further studies to elucidate the relationship between diabetes and other chronic medical conditions on physical, mental and social domains of HRQoL in the general population are clearly needed.

The primary purpose of this study was therefore to evaluate the impact of diabetes and co-existing chronic medical conditions on the physical, mental and social domains of HRQoL in the general population. We also sought to determine if the impact of diabetes and co-existing chronic medical conditions on HRQoL would be additive, synergistic or subtractive. For these purposes, we measured HRQoL using both the SF-36 and the SF-6D. The SF-36 is a comprehensive, generic HRQoL measure that has been extensively validated and allows comparison of HRQoL in subjects with various chronic medical conditions. The SF-6D is a single index utility score derived from the SF-36 which measures health preference, and which is therefore suitable for pharmacoeconomic analyses to assist healthcare resource allocation. We sought to determine the extent to which the SF-6D would reflect the multidimensional information provided by the SF-36.

## Methods

### Study design

We analyzed data from a cross-sectional, population-based disproportionately stratified sample of ethnic Chinese, Malays and Indians listed in the 1996 electoral register for the Bishan-Toa Payoh district (representative of the general population) in Singapore from April 1998 to January 1999, details of which have been reported previously [25] and are summarized here. Participants completed either the UK English or Chinese (Hong Kong) SF-36 and the Family Functioning Measure. Eligibility criteria were: age 21 to 65 years on 1^st ^January 1998 and ability to read a newspaper in English or Chinese; Chinese, Malay or Indian ethnicity as reflected in the National registration identification card, which reflects parental ethnicity; and status as free-living adults in the community. Persons older than 65 years of age were excluded because of low literacy rates in this age group in Singapore [26]. Fieldworkers visited eligible subjects at their house within 7 days after an introductory letter was sent, invited them to self-administer the SF-36, checked returned questionnaires for completeness and obtained information on ethnicity, socioeconomic status and chronic medical conditions (including diabetes, hypertension, heart disease, lung diseases, musculoskeletal illnesses and mental illnesses) and other potential determinants of HRQoL through a structured interview. Participants were asked to indicate either "yes" or "no" on a list of chronic medical conditions including diabetes, high blood pressure, heart disease, etc.

### Instruments

#### The Short-Form 36 Health Survey (SF-36)

The SF-36 measures perceived health in the areas of physical functioning (PF), role-physical (RP), bodily pain (BP), general health (GH), vitality (VT), social functioning (SF), role-emotional (RE), and mental health (MH), with higher scores (range 0–100) reflecting better perceived health. The UK English [27] and Chinese (Hong Kong) [28] SF-36 with 4-week recall and metric units of measurement were used in this study (previously validated for use in Singapore) [25].

#### The SF-6D

The SF-6D is a six-dimensional health classification system assessing physical functioning (PF), role limitations (RL), social functioning (SF), pain (PN), mental health (MH), and vitality (VT), with 4 to 6 levels per dimension [29,30]. A SF-6D "health state" is defined by selecting one level from each dimension. For example, perfect health on the SF-6D would be represented by the health state 111111. A total of 18,000 health states are thus defined. All responders to the original SF-36 questionnaire can be assigned an SF-6D score provided the items used to construct the SF-6D are completed [29,30]. The SF-6D preference-based measure can be regarded as a continuous variable scored on a 0.29 to 1.00 scale, with 0.29 corresponding to the worst health state (i.e. all dimension being at the worst level) and 1.00 corresponding to "full health" (i.e. all dimensions being at full functional level).

#### Family Functioning Measure

The Family Functioning Measure is a 3-item Likert scale assessing the quality of interactions among family members [31], with higher scores (range 0–100) reflecting better family functioning, and has been validated for use in Singapore [32].

### Statistical analyses

Data from participants completing English and Chinese SF-36 versions were pooled to increase the power and representativeness of our study. This approach was supported by previous work demonstrating equivalence of these SF-36 [33] and SF-6D [34] versions in this same study sample. Data were entered into an Excel spreadsheet (Microsoft Corporation, Redmond, Washington) and analyzed using SPSS (SPSS Inc., Chicago, Illinois) software. SF-6D index scores were calculated using a utility function derived from the United Kingdom general population [29], which is currently the only published SF-6D scoring algorithm. Participants with missing scale scores were excluded listwise from analysis.

To study the influence of diabetes and other chronic medical conditions on HRQoL, we developed 9 multiple linear regression models, with each SF-36 scale and the SF-6D index score being the dependent variable for a separate model. Each model was built in 3 stages. The first stage assessed the effect of diabetes on HRQoL while adjusting for the influence of sociodemographic factors. The second stage assessed the independent effects of diabetes and a second chronic medical condition on HRQoL while adjusting for the influence of sociodemographic factors. The third stage additionally assessed any interactions between diabetes and this second chronic medical condition on HRQoL. Thus, each model studied the effect of diabetes and one other chronic medical condition. The absence of a significant interaction term would suggest that the influence of two chronic medical conditions on HRQoL scores was additive. The presence of a significant interaction term would suggest that a synergistic or subtractive relationship existed, the former if the coefficient for the interaction term was negative, the latter if the coefficient was positive (since changes in HRQoL scores in the presence of chronic medical conditions were expected to be negative). Ethnicity was coded using dummy variables. Educational level was used as proxy for socioeconomic status. In anticipation that if the number of subjects with a given chronic medical condition was less than 50, the numbers of subjects with this medical condition and diabetes would be very small and not amenable to meaningful interpretation, two chronic medical conditions, stroke and cancer, were thus excluded from analysis because of small numbers of subjects reporting these conditions (n<50). Two other chronic medical conditions were subsequently excluded from analysis because of small numbers of subjects with diabetes and these conditions (diabetes and lung diseases: n = 27 and diabetes and mental illnesses: n = 9).

## Results

### Characteristics of study subjects

Complete data were available from 5,224 of 5,420 subjects in the study, with 196 (3.6%) subjects excluded from analysis (110 because of missing demographic information and 86 because of missing responses to the SF-36). Table 1 shows subjects' characteristics and distribution of SF-36 scales and SF-6D index scores. Approximately 6% of subjects suffered from diabetes and two-fifths of subjects reported at least 1 chronic medical condition. Prevalence of co-existing conditions in subjects with diabetes ranged from 2.9% (mental illnesses) to 37.2% (hypertension).

**Table 1:** Characteristics of subjects and distribution of SF-36 scales and SF-6D index scores.

Subject characteristics (N = 5,224)	N (%) unless specified otherwise
Mean (SD) age in years	40.3 (11.52)
Female gender	2,555 (48.9)
Ethnicity	
Chinese	2,558 (49.0)
Malay	1,189 (22.8)
Indian	1,477 (28.3)
Housing type	
Lower cost public housing	388 (7.4)
Public housing	4,647 (89.0)
Private housing	188 (3.6)
Years of education	
≤ 6	1,266 (24.2)
7–10	2,682 (51.3)
>10	1,276 (24.4)
Marital status	
Married	3,686 (70.6)
Single	1,272 (24.3)
Divorced/Separated	136 (2.6)
Widow	130 (2.5)
Working	4,022 (75.0)
Smoking	1,045 (20.0)
Presence of acute medical conditions*	3,493 (66.9)
Presence of chronic medical conditions	
Diabetes mellitus	309 (5.9)
Hypertension	557 (10.7)
Heart disease	125 (2.4)
Stroke	32 (0.6)
Lung diseases^‡^	278 (5.3)
Cancer	35 (0.7)
Musculoskeletal illnesses^‡^	1,389 (26.6)
Mental illness^‡^	136 (2.6)
Prevalence of co-existing chronic medical conditions in people with diabetes mellitus	
Hypertension	115 (37.2)
Heart disease	46 (14.9)
Lung diseases^‡^	27 (8.7)
Musculoskeletal illnesses^‡^	107 (34.6)
Mental illnesses^‡^	9 (2.9)
Mean (SD) Family Functioning Measure scores	10.3 (2.75)
Mean (SD) SF-36 scores^†^	
Physical functioning (PF)	80.3 (23.19)
Role-physical (RP)	80.5 (32.94)
Bodily pain (BP)	78.3 (21.73)
General health (GH)	68.6 (17.21)
Vitality (VT)	63.8 (16.98)
Social functioning (SF)	80.6 (20.48)
Role-emotional (RE)	79.2 (34.98)
Mental health (MH)	72.4 (16.70)
Mean (SD) SF-6D index scores^†^	0.77 (0.130)

* Acute medical conditions were self-limiting illnesses and injuries in the preceding one month that might influence SF-36 scores and included acute illnesses (e.g. upper respiratory tract infections, gastroenteritis and headaches), injuries or poor sleep. ^† ^Mean scores adjusted for the influence of diabetes mellitus, other chronic medical conditions (and their interactions), socioeconomic status and other factors. ^‡ ^Lung diseases included asthma and other lung diseases; musculoskeletal illnesses included rheumatism, back pain and other bone or muscle illness; mental illness included depression, anxiety disorder and schizophrenia.

### The influence of chronic medical conditions on SF-36 scores

The influence of chronic medical conditions on individual SF-36 scales is presented in Table 2. Before adjusting for selected sociodemographic variables known to influence HRQoL (i.e. age, gender, ethnicity and years of education), subjects with self-reported chronic medical conditions (diabetes, hypertension, heart disease and musculoskeletal illnesses) generally reported lower scores for most SF-36 scales when compared to subjects without these conditions, indicating poorer HRQoL. After adjusting for the influence of these sociodemographic variables, chronic medical conditions continued to influence HRQoL, generally to a lesser degree than before adjustment. The influences of heart disease and musculoskeletal illnesses on SF-36 scores were of a similar magnitude and were larger than the influence of diabetes or hypertension.

**Table 2:** Influence of diabetes mellitus and other chronic medical conditions on SF-36 scales.

	Unadjusted differences in mean scores								
	PF	RP	BP	GH	VT	SF	RE	MH	SF-6D

No chronic medical condition^† ^ (n = 3155)	82.0 (22.61)	81.9 (32.16)	80.4 (21.20)	70.0 (16.62)	64.8 (16.43)	81.5 (20.12)	77.5 (36.00)	73.1 (16.35)	0.79 (0.123)
									
Diabetes mellitus only (n = 309)	-7.2	-7.3	-5.8	-3.5	-1.3	-1.8	0.7	0.2	-0.06
									
Hypertension only (n = 557)	-4.3	-4.1	-3.4	-3.3	-0.5	0.5	1.0	0.3	-0.05
									
Heart disease only (n = 125)	-9.5	-10.1	-7.3	-5.2	-3.6	-0.8	-4.7	-0.3	-0.09
									
Musculoskeletal illnesses only^‡ ^ (n = 1389)	-4.7	-3.6	-6.9	-4.4	-3.1	-2.7	-0.8	-2.5	-0.08
	Adjusted differences in mean scores due to medical condition^§^								

	PF	RP	BP	GH	VT	SF	RE	MH	SF-6D

No chronic medical condition^† ^ (n = 3155)	0.0	0.0	0.0	0.0	0.0	0.0	0.0	0.0	0.00
									
Diabetes mellitus only (n = 309)	-2.9 *	-3.7	-1.8	-2.36 *	-1.3	-0.1	0.3	-0.2	-0.03 **
									
Hypertension only (n = 557)	-2.6 *	-3.1 *	-1.5	-1.6	0.1	0.6	-1.0	0.1	-0.03 ***
									
Heart disease only (n = 125)	-5.0 *	-6.5 *	-3.5	-4.0 *	-3.9 *	0.9	-5.9	-1.0	-0.06 ***
									
Musculoskeletal illnesses only^‡ ^ (n = 1389)	-3.8 ***	-3.1 **	-6.4 ***	-3.9 ***	-2.9 ***	-3.1 ***	-3.4 **	-2.6 ***	-0.08 ***

^†^Mean (SD) scores

^‡^Musculoskeletal illnesses included rheumatism, back pain and other bone or muscle illnesses.

^§^Multiple linear regression, adjusted for influence of ethnicity, age, gender and years of education.

*p < 0.05, **p < 0.01, ***p < 0.001

Abbreviations: PF = Physical Functioning, RP = Role-Physical, BP = Bodily Pain, GH = General Health, VT = Vitality, SF = Social Functioning, RE = Role -Emotional, MH = Mental Health

### The influence of chronic medical conditions on SF-6D scores

The influence of chronic medical conditions on SF-6D scores is also presented in Table 2. Subjects with chronic medical conditions similarly reported lower unadjusted mean SF-6D scores. After adjusting for known determinants of HRQoL, lowering of SF-6D scores persisted for all chronic medical conditions, though again to a smaller magnitude. The influences of heart disease and musculoskeletal illnesses on SF-6D scores were of a similar magnitude and were again larger than the influence of diabetes or hypertension.

### The influence of diabetes mellitus and co-existing chronic medical conditions on SF-36 scores

Characteristics of subjects with diabetes, with and without other co-existing chronic medical conditions, are shown in Table 3. Subjects with diabetes only (i.e. no co-existing chronic medical conditions) were generally younger and more likely to be male. There were more Indians with diabetes and co-existing heart disease or musculoskeletal illnesses and more Chinese with diabetes and co-existing hypertension. The distribution of years of education completed was fairly similar in the various ethnic groups.

**Table 3:** Characteristics of subjects with diabetes, with and without other chronic medical conditions.

	Co-existing chronic medical conditions among subjects with diabetes			
Characteristics	No co-existing chronic medical conditions (n = 115)	Hypertension (n = 115)	Heart disease (n = 46)	Musculoskeletal illnesses^† ^ (n = 107)

Mean age (years)	48.1 (10.52)	53.7 (8.46)	55.5 (7.95)	53.2 (8.68)
				
Male (%)	64.3	56.5	71.7	54.2
				
Ethnicity (%)				
Chinese	23.5	40.0	21.7	36.4
Malays	27.8	22.6	21.7	17.8
Indians	48.7	37.4	56.5	45.8
				
Questionnaire language (English) (%)	86.1	73.9	91.3	76.6
				
Education (%)				
≤ 6 years	36.5	44.3	30.4	41.1
7–10 years	50.4	42.6	56.5	48.6
> 10 years	13.0	13.0	13.0	10.3

The impact of co-existing chronic medical conditions on adjusted SF-36 scores of subjects with diabetes is presented in Table 4. In general, after adjusting for known determinants of HRQoL, the presence of concurrent hypertension, heart disease and musculoskeletal illnesses further reduced SF-36 scores in subjects with diabetes. For example, subjects with diabetes and co-existing hypertension or musculoskeletal illnesses experienced further lowering of physical functioning scores by 2.3 and 3.7 points, respectively. The influence of two chronic medical conditions on SF-36 scores was generally additive, in that statistically significant interaction terms were not present for most comparisons. There were some exceptions in which subtractive effects were observed, mainly clustered within subjects with diabetes and co-existing heart disease (physical functioning, role-physical, social functioning and role-emotional scales) or musculoskeletal illnesses (bodily pain and social functioning scales).

**Table 4:** Multiple linear regression models of the influence of diabetes mellitus and a second chronic medical condition (i.e. hypertension, heart disease or musculoskeletal illnesses) adjusted for ethnicity, age, gender and years of education on SF-36 and SF-6D scores.

	Co-existing chronic medical conditions among subjects with diabetes			
	No co-existing chronic medical conditions (n = 115)	Hypertension (n = 115)	Heart disease (n = 46)	Musculoskeletal illnesses^† ^ (n = 107)

Physical Functioning				
Differences in scale scores due to				
Diabetes mellitus	-2.9*	-2.4	-3.5*	-2.8*
2nd chronic medical condition‡	na	-2.3*	-7.9**	-3.7***
Interaction termξ	na	ns	12.46**	ns
Role-Physical				
Differences in scale scores due to				
Diabetes mellitus	-3.7	-3.1	-5.2*	-3.6
2nd chronic medical condition‡	na	-2.8	-12.5**	-3.1**
Interaction termξ	na	ns	20.1**	ns
Bodily Pain				
Differences in scale scores due to				
Diabetes mellitus	-1.8	-1.6	-1.5	-3.8*
2nd chronic medical condition‡	na	-1.3	-3.1	-6.9***
Interaction termξ	na	ns	ns	7.0**
General Health				
Differences in scale scores due to				
Diabetes mellitus	-2.3*	-2.0	-1.9	-2.2*
2nd chronic medical condition	na	-1.3	-3.5*	-3.9***
Interaction termξ	na	ns	ns	ns
Vitality				
Differences in scale scores due to				
Diabetes mellitus	-1.3	-1.4	-0.9	-1.3
2nd chronic medical condition‡	na	0.3	-3.7*	-2.9***
Interaction termξ	na	ns	ns	ns
Social Functioning				
Differences in scale scores due to				
Diabetes mellitus	-0.1	-0.2	-1.3	-1.9
2nd chronic medical condition^‡^	na	0.6	-2.5	-3.4***
Interaction term^ξ^	na	ns	10.2*	5.4*
Role-Emotional				
Differences in scale scores due to				
Diabetes mellitus	0.3	-2.8	-0.9	0.4
2nd chronic medical condition^‡^	na	-2.7	-11.8**	-3.4**
Interaction term^ξ^	na	9.9*	16.8*	ns
Mental Health				
Differences in scale scores due to				
Diabetes mellitus	-0.2	-1.7	-0.1	-0.1
2nd chronic medical condition^‡^	na	-0.5	-0.9	-2.6***
Interaction term^ξ^	na	5.1*	ns	ns
SF-6D				
Differences in scale scores due to				
Diabetes mellitus	-0.03**	-0.02*	-0.02*	-0.02**
2nd chronic medical condition^‡^	na	-0.03***	-0.06***	-0.08***
Interaction term^ξ^	na	ns	ns	ns

^†^Musculoskeletal illnesses included rheumatism, back pain and other bone or muscle illnesses.

^‡^Second chronic medical condition refers to the chronic medical condition other than diabetes mellitus listed in the relevant column (e.g. hypertension, heart disease or musculoskeletal illnesses).

^ξ ^Interaction term for diabetes mellitus and second chronic medical condition.

*p < 0.05, **p < 0.01, ***p < 0.001, ns denotes statistically insignificant.

### The influence of diabetes mellitus and co-existing chronic medical conditions on SF-6D scores

The influence of diabetes and co-existing chronic medical conditions on SF-6D scores is presented in Table 4. Subjects with diabetes and other co-existing chronic medical conditions reported lower unadjusted SF-6D scores than subjects with diabetes only. After adjustment for known determinants of HRQoL, the influence of co-existing chronic medical conditions on SF-6D scores persisted. As before, subjects with diabetes and co-existing heart disease or musculoskeletal illnesses reported the greatest impairment in HRQoL. Diabetes and other co-existing chronic medical conditions, including heart disease, reduced SF-6D scores in an additive fashion.

## Discussion

In this multiethnic, population-based study, we found that subjects with diabetes experienced lowering of HRQoL as compared to subjects without diabetes. The presence of other chronic medical conditions in subjects with diabetes led to further lowering of HRQoL, the effect of which was generally additive. Our findings further underscore the importance of preventing and treating complications of diabetes to prevent further deterioration in HRQoL among subjects with diabetes, and also highlight the need to identify factors that may be modulated to improve HRQoL in these subjects.

Our findings are important for several reasons. First, this is the first study showing that the combination of diabetes and a second chronic medical condition may adversely affect the mental domains of HRQoL as measured by the vitality, social functioning and mental health scales of the SF-36 (previous studies having focused on the physical domains of HRQoL) [23,24]. Second, it is reassuring that the effect of diabetes and a second chronic medical condition on HRQoL was in general additive rather than synergistic. Third, given that subjects were drawn from the general population, it is likely that our findings can be readily generalized to the population at large, especially given that this study was conducted in a multiethnic Asian population with one of the highest diabetes prevalence rates in the world [35].

We found that the effect of diabetes on HRQoL was generally mild, with greater impact on the SF-36 scales measuring physical (physical functioning, role-physical, bodily pain, general health, role-emotional (in this study sample)) relative to mental health components (vitality, social functioning and mental health). This was not surprising, given that our subjects were recruited from the general population and were therefore likely to have less severe illness than subjects in hospital or clinic-based studies. Further, other studies have shown that impact of diabetes on HRQoL is intermediate, relative to other chronic medical conditions [14]. For example, Egede [9] reported that the risk of functional disability was lower in subjects with diabetes than subjects with major depression (odds ratio (OR): 2.42 vs 3.00), while Otiniano et al. [23] found that the risk of disabilities in ADL were lower in subjects with diabetes than those with strokes (OR: 2.80 vs 5.55).

We also found that with the exception of subjects with diabetes and heart disease, the presence of co-existing chronic medical conditions in subjects with diabetes generally resulted in further significant lowering of HRQoL. Our results are important because they demonstrate that the impact of these co-existing chronic medical conditions in diabetes is not only in increasing healthcare costs [36,37] and mortality [38] but also in increasing the physical and psychosocial burden of diabetes. Given that complications of diabetes constitute the majority of chronic medical conditions commonly present in subjects with diabetes, our findings further underscore the importance of preventing and treating complications of diabetes, and also highlight the need to identify factors that may be modulated to improve HRQoL in these subjects.

In our study, the relationships between diabetes and other chronic medical conditions on HRQoL were largely additive. This is in contrast with the study by Gaynes et al. [19], where synergistic effects between depression and diabetes on HRQoL were observed. Instead, we found in general an additive effect with several comparisons showing a subtractive effect on HRQoL (especially in subjects with diabetes and co-existing heart disease). There are several possible explanations for the presence of subtractive effects. One possible explanation is the presence of response shift related to the specific diseases involved. In this study, the subtractive effects were clustered among subjects with diabetes and co-existing heart disease. Diabetes (in particular Type 2 diabetes) is a well-recognized cardiovascular risk factor with subjects with diabetes commonly developing heart disease in the 5^th ^or 6^th ^decade of life [40], often many years after the diagnosis of diabetes [41] (although heart disease may on occasion be present at or before diagnosis) [42,43]. In contrast, hypertension typically precedes or occurs soon after diagnosis in subjects with Type 2 diabetes, with up to 50% of subjects with diabetes presenting with hypertension at the time of diagnosis [44]. Hence, adaptation to the underlying diabetes may have led to response shift occurring in subjects with diabetes and co-existing heart disease but not with hypertension. A second plausible explanation for this observation is a healthy-responder effect. This would occur if subjects with diabetes and co-existing heart disease were more likely to have passed away or were too sick to participate in the study. Thus the subset of subjects with diabetes and co-existing heart disease who did participate in this study would reflect the healthier end of the spectrum, leading to the observed effect of higher scores for respondents with the two co-existing chronic medical conditions. A third possible explanation is that some conditions are so similar in their effects on HRQoL that having more than one such condition is not particularly problematic to the individual. For example, in our study, subjects with diabetes alone and subjects with heart disease alone experienced lower scores on the physical functioning scale of the SF-36; if these two conditions exerted a similar effect on HRQoL, then a subtractive effect would be expected, as was indeed observed among subjects with diabetes and co-existing heart disease.

A secondary objective of this study was to understand the extent to which the single index SF-6D captured information from the multidimensional SF-36. We found that in general, the impact of co-existing chronic medical conditions on the SF-36 was well-reflected in the SF-6D. However, the subtractive effects of diabetes and co-existing heart disease on SF-36 scores were not reflected in SF-6D scores. This suggests that there is some inevitable loss of information associated with a reduction from a multi-dimensional scale to a unidimensional scale. Hence, more studies are needed to evaluate the adequacy of the SF-6D as a summary measure of the SF-36.

We recognize several limitations of this study. First, identification of chronic medical conditions was based on self-report which may not be as accurate as physician diagnoses. However, reliability of self-report has been found to be acceptable for conditions that required medical or laboratory diagnostic procedures, including diabetes [45-47]. Second, although we captured information on subjects with diabetes and co-existing mental illnesses or lung diseases, we had to exclude these because of the small number of subjects. Finally, in this study we did not differentiate between subjects with Type 1 and Type 2 diabetes. However, given that more than 90% of subjects with diabetes are Type 2, this is not likely to affect our findings.

## Conclusions

In conclusion, in this large, multiethnic, population-based study, we found that subjects with diabetes experienced lowering of HRQoL as compared to subjects without diabetes. The co-existence of other chronic medical conditions in subjects with diabetes led to further lowering of HRQoL, the effect of which was generally additive. Finally, we found that the SF-6D is a reasonably good summary measure of the SF-36 although more studies are needed to confirm this observation.

## List of abbreviations

ADL – Activities of Daily Living, DM – Diabetes mellitus, HD – Heart disease, HRQoL – Health-related quality of life, HTN – Hypertension, MS – Musculoskeletal illnesses, OR – Odds ratio, SF-36 – Medical Outcomes Short-Form 36.

## Authors' contributions

JT conceived of the study, and participated in its design and coordination. HL Wee and YB Cheung participated in the design of the study and performed the statistical analysis. SC Li and KY Fong participated in the design of the study and its coordination. All authors read and approved the final manuscript.
